# Pharmacogenomic Research in South Africa: Lessons Learned and Future Opportunities in the Rainbow Nation

**DOI:** 10.2174/187569211796957575

**Published:** 2011-09

**Authors:** Louise Warnich, Britt I Drögemöller, Michael S Pepper, Collet Dandara, Galen E.B Wright

**Affiliations:** 1 Department of Genetics, Stellenbosch University, Stellenbosch, South Africa; 2 Department of Immunology, Faculty of Health Sciences, University of Pretoria, Pretoria, South Africa; 3 Department of Genetic Medicine and Development, Faculty of Medicine, University of Geneva, Geneva, Switzerland; 4 Division of Human Genetics, University of Cape Town, Cape Town, South Africa

**Keywords:** Disease burden, ELSI, genomic technologies, global health, personalized medicine, pharmacogenomics, population genetic diversity, South Africa.

## Abstract

South Africa, like many other developing countries, stands to benefit from novel diagnostics and drugs developed by pharmacogenomics guidance due to high prevalence of disease burden in the region. This includes both communicable (e.g., HIV/AIDS and tuberculosis) and non-communicable (*e.g*., diabetes and cardiovascular) diseases. For example, although only 0.7% of the world’s population lives in South Africa, the country carries 17% of the global HIV/AIDS burden and 5% of the global tuberculosis burden. Nobel Peace Prize Laureate Archbishop Emeritus Desmond Tutu has coined the term Rainbow Nation, referring to a land of wealth in its many diverse peoples and cultures. It is now timely and necessary to reflect on how best to approach new genomics biotechnologies in a manner that carefully considers the public health needs and extant disease burden in the region. The aim of this paper is to document and review the advances in pharmacogenomics in South Africa and importantly, to evaluate the direction that future research should take. Previous research has shown that the populations in South Africa exhibit unique allele frequencies and novel genetic variation in pharmacogenetically relevant genes, often differing from other African and global populations. The high level of genetic diversity, low linkage disequilibrium and the presence of rare variants in these populations question the feasibility of the use of current commercially available genotyping platforms, and may partially account for genotype-phenotype discordance observed in past studies. However, the employment of high throughput technologies for genomic research, within the context of large clinical trials, combined with interdisciplinary studies and appropriate regulatory guidelines, should aid in acceleration of pharmacogenomic discoveries in high priority therapeutic areas in South Africa. Finally, we suggest that projects such as the H3Africa Initiative, the SAHGP and PGENI should play an integral role in the coordination of genomic research in South Africa, but also other African countries, by providing infrastructure and capital to local researchers, as well as providing aid in addressing the computational and statistical bottlenecks encountered at present.

## 1 INTRODUCTION

Situated at the tip of the African continent, South Africa is home to an eclectic array of cultures, illustrated by the eleven official languages recognized in the country. Today, South Africa is acknowledged for its impressive political change, progress and development [1], but the country has a complex history of free and forced migration and immigration, oppression and liberation. In an assessment of South Africa’s health status and systems, Coovadia *et al.* [2] point out that the challenges that South Africa is currently facing are deeply rooted in this unique history. 

At present South Africa faces a high burden of both communicable and non-communicable diseases, high maternal and child mortality, as well as injury and violence related deaths [2]. Although only 0.7% of the world’s population lives in South Africa, the country carries 17% of the global Human Immunodeficiency Virus/Acquired Immunodeficiency Syndrome (HIV/AIDS) burden and 5% of the global tuberculosis (TB) burden [3, 4]. HIV/AIDS is also one of the leading causes of maternal and child deaths and South Africa is one of only twelve countries for which the mortality rate of children has increased since the Millennium Development Goals baseline in 1990 [5, 6]. The most prevalent non-communicable diseases in South Africa are cardiovascular diseases, diabetes mellitus, respiratory diseases, cancers and neuropsychiatric disorders [7]. The rise in these diseases is associated with an increase in risk factors due to demographic changes over the past few years, and their distribution largely reflects socioeconomic disparities between communities [7]. This high burden of infectious and chronic diseases results in a health system that is continuously under-resourced. Accordingly, South Africa’s per capita health burden is the highest of any middle-income country in the world and its health outcomes are often worse than lower income countries [2, 8]. 

Interventions aimed at addressing the burden of HIV/AIDS, TB and other communicable and non-communicable diseases are multi-faceted, but rely heavily on prevention and treatment, which in turn entails drug therapy. Firstly, it is necessary that drug regimens are accessible to all. Although some limitations still exist, free anti-retroviral therapy (ART) has been available in the public health system since 2003, and has dramatically improved the survival of HIV-infected patients [3]. With a public health system that is already under enormous pressure, it is therefore imperative that drug treatment plans be efficient and cost-effective. Treatment, however, is frequently associated with toxicity, which impacts on its efficacy and costs [9]. It is well known that adverse drug reactions (ADRs) are responsible for a notable number of hospitalizations, and fatal ADRs are among the leading causes of death in developed countries [10, 11]. In South Africa, ADRs are reported to occur in 14% of hospitalized patients, as opposed to the 6.7% reported internationally, with a five to ten times higher fatality rate [12]. This study also showed that the HIV/AIDS and TB epidemics have a significant impact on the epidemiology of ADRs in South Africa, often due to polypharmacy. Not only do ADRs contribute to the financial burden on the health system, but they also contribute to poor patient compliance, with the subsequent emergence of drug resistant pathogens, and the continuation of epidemics [13-15]. These data hold major implications for the delivery of health care services in South Africa, especially when considering chronic illnesses, such as HIV/AIDs, that require lifelong treatment. 

Pharmacogenomic applications (as seen in the broader context of the field, [16]) hold the promise of using genome-based technologies to improve health by the prevention or effective treatment of disease. Even in the developing world, pharmacogenomics can serve to improve a country’s “ability to respond to local disease threats” and to “make better policy decisions regarding the most optimal interventions within their own health care delivery systems” [16]. Specifically, the implementation of pharmacogenomics in the treatment of HIV/AIDS should be a priority in South Africa. Unfortunately, very few clinically validated ART pharmacogenetic markers are available, and none, to our knowledge, have been validated in the context of South African populations and treatment regimes. Currently no pharmacogenetic test is indicated in the South African ART guidelines [17]. Furthermore, some of the available bio-markers may not have so much utility in the South African setting. For example, although HLA-B*5701 screening to identify patients who are at risk to develop abacavir hypersensitivity syndrome has been shown to be cost-effective and has been included in treatment guidelines and drug labels in the United States (US) and Europe, this allele seems to be rare in certain African populations [18-20]. Additionally, in South Africa, abacavir is only included in the guidelines for treatment of infants and children and is not one of the most frequently prescribed drugs. Therefore, studies examining the effect of variants in genes involved in the metabolism, transport and absorption of drugs included in the current first or second line ART regimen in South Africa [17], such as *CYP2B6* that is involved in the metabolism of efavirenz and nevirapine, are required. 

Despite the fact that pharmacogenomics was specifically highlighted in The National Biotechnology Strategy for South Africa [21] and that the development of technology platforms for pharmaceutical industries is advocated in a Ten Year Innovation Plan by the South African Department of Science and Technology (DST) [21], new developments in this field within the country have been slow. For example, the large scale genotyping project to profile the South African populations has not been fully realized yet, neither has a central bio-repository or database been established [22]. 

The current genomic challenge is to ascertain the genetic diversity in our understudied populations, to understand genotype-environment interactions, and to translate this knowledge into clinical applications that can be applied in public health care. This report provides an overview of the populations of South Africa and demonstrates the unique genetic and pharmocogenetic profiles of some of these populations as revealed by a limited number of studies. The paper also discusses the ethical, social and legal frameworks that are in place or are being developed, and points to some of the opportunities and challenges that can impact on the realization of the full benefits of pharmacogenomics to improve health in South Africa. 

## 2 THE RAINBOW NATION

Nobel Peace Prize Laureate Archbishop Emeritus Desmond Tutu has coined the term Rainbow Nation, referring to a land of wealth in its many diverse peoples and cultures. It is now timely and necessary to reflect on how best to approach new genomics biotechnologies in a manner that carefully considers the public health needs and extant disease burden in the region. 

The interesting and diverse history of South Africa is reflected in the genomes of the nearly 50 million individuals residing within the country [4]. The first modern humans in southern Africa were the Khoisan-speakers and Bantu-speakers, who arrived approximately 20,000 and 4,000 years ago, respectively [23]. A few thousand years later, European settlers landed in South Africa, the most noteworthy of which were the Dutch in 1652 and the British around the start of the 19^th^ century. Accompanying these early settlers were slaves, political prisoners, laborers and traders, originating predominantly from East Africa and Asia [24, 25]. This varied history has resulted in South African populations which range from ancient and genetically diverse, to highly admixed, to recent and homogenous. Broadly speaking, the most recent South African population census has divided the Rainbow Nation into four population groups, namely Africans, Whites (Caucasians), Coloureds (Mixed Ancestry) and Indians/Asians, which constitute 79.4%, 9.2%, 8.8% and 2.6% of the total population, respectively [4]. These broad population census definitions, however, only hint at the number of diverse ethnic groups residing within South Africa. 

The black African people of South Africa are descendants of ancient and diverse populations. Africa is believed to be the site of origin of the earliest reported humans and African populations are consequently renowned for their considerable genetic diversity, not only due to their age, but also because of their exposure to varying diets, climates, infectious diseases and geography [26, 27]. Southern African populations, specifically, have been shown to exhibit the highest level of genetic diversity and the greatest within-population diversity. For example, the differences observed between genomes of two southern African Khoisan individuals living within walking distance from one another were reported to be greater than the differences observed between Asian and European individuals living on different continents [28]. 

**Fig. (1):**
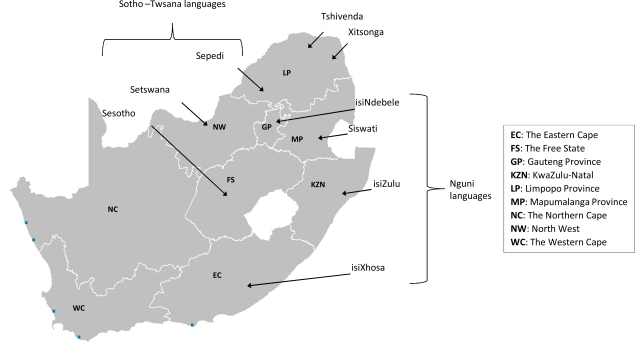
The approximate historical location of the different African (Bantu) linguistic population groups within South Africa (adapted from [33]).

Individuals with African ancestry that reside in South Africa today have reportedly arisen from two main groups: the Bantu from West Central Africa and the Khoisan, who are one of the most ancient populations, from eastern Africa [23, 29]. Although the Bantu and Khoisan are discernable from one another, over time, admixture has occurred between these two groups [25, 30, 31], which is reflected in the clicks present in some of the Bantu languages spoken in South Africa (see Table **1** for the official South African languages). Almost a quarter (23.8%) of the population indicated isiZulu as their home language in the 2001 Census [32]. The second largest home language group was isiXhosa (17.6%), while the five least spoken home languages (Sesotho, Xitsonga, siSwati, Tshivenda and isiNdebele) collectively comprised 19%. A study performed by Lane *et al*. [33], examining autosomal short tandem repeats and mitochondrial DNA (mtDNA) variation, showed that although there is a high level of within-group genetic diversity present in the different Bantu populations, the level of relatedness between the groups suggests a common ancestral population. Even so, the different linguistic groups can be divided up into sub-groups, according to the examined autosomal and mtDNA variation, which roughly correspond to their historical geographic location within South Africa (Fig. **1**).

While the Africans residing within South Africa represent ancient populations, the South African Coloured or Mixed Ancestry population, which reportedly exhibits the highest level of admixture worldwide [34], is estimated to have arisen only about 350 years ago [25]. The term Coloured, which is the preferred term used by the members of the population themselves [25], was only introduced in 1808 with the abolishment of slavery [35]. The origin of this unique population group can largely be attributed to South Africa’s position in major trade routes, which attracted individuals from all over the world. Genetic studies examining variation on a genome-wide level have shown that this population group has ancestry contributions from at least four different sources, namely Xhosa, Khoisan, European and Asian [25, 34, 36], the largest of which stems from the Khoisan [25, 30, 36, 37]. Considerable variation, however, can exist between individuals regarding ancestry proportions [34, 36]. Additionally, recent studies have shown that there was a gender biased gene flow, which is in accordance with the history of South Africa. This gene flow suggests that the majority of the Coloured population arose from male dominated European settlers and female slaves/natives [25, 37]. 

Whereas African and Coloured populations represent genetically diverse and admixed population groups, Caucasian and Indian South Africans are relatively homogenous in nature. This being said, although most of South African Caucasian and Indian ancestry has been identified as originating from European or Indian ancestors, small amounts of gene flow from Africans have been detected in both populations [30, 38, 39]. Focusing on the Caucasians, one of the earliest groups to take up residence in South Africa were Dutch East India Company settlers, who set up a refreshment station in the Cape in 1652. This group of individuals gave rise to the Afrikaner population, which is homogenous as a result of a small founding population, in combination with a rapid population increase [24]. The homogeneity of this population group is reflected in the high level of linkage disequilibrium (LD) observed [40] and the high frequency of rare disorders, such as variegate porphyria and familial hypercholesterolemia [24]. Similarly, the South African Indian population has a relatively high incidence of insulin resistance, which is coupled to increased risks for type 2 diabetes and ischemic heart disease [41]. The observed homogeneity in the Indian population may in part be attributed to strict religious customs, which have traditionally prevented marriage outside this ethnic group, and is reflected in the diminished mtDNA variation in these individuals, when compared to Indians living in Nepal [38]. 

Although genetic analyses of the diverse populations living in South Africa may prove challenging, there are also several unique benefits that these populations may offer to future genomic studies. Thus, the genetic diversity, homogeneity and admixture of these population groups should be utilized to elucidate the genetic factors contributing to complex disorders and traits, including drug response. 

## 3 GENETIC VARIATION IN PHARMACOGENETICALLY RELEVANT GENES (PHARMACOGENES) IN SOUTH AFRICAN POPULATIONS

Current knowledge on the genetic diversity of South Africa’s diverse populations, and specifically pharmacogenetic diversity, is based on a limited number of studies that have focused on candidate genes, using relatively small sample sizes. In the following section we will highlight a few of these studies. The data obtained thus far indicate that our populations demonstrate unique genetic profiles that include novel and rare variants, with allele frequencies often differing from each other and other African populations. It is not within the scope of this paper, however, to provide an extensive and complete overview of all studies that have been performed to date. Examples of frequencies of the most clinically important pharmacogene variants detected in South African populations are shown in Table **2**. For a more expanded comparison of allelic variation in 12 genes that influence drug treatment, using two South African populations, the Xhosa and the Coloured, the reader is referred to a recent study by Ikediobi *et al.* [42].

### 3.1 Cytochrome P450 (*CYP*) Genes

To date, most studies on important pharmacogenes in South African populations have been conducted on genes encoding the major drug metabolizer family, the Cytochrome P450 (CYP) enzymes. The CYP enzymes play an important role in the Phase 1 metabolism of endogenous and exogenous compounds, including the majority of clinically administered drugs. Specific genetic polymorphisms or haplotypes in these genes define specific alleles [43]. Different alleles are often known to have different effects on enzyme activity and individuals are classified as poor (PM), extensive (EM), intermediate (IM) or ultrarapid (UM) metabolizers for specific enzymes. 

#### CYP2B6

The CYP2 family includes some of the most important metabolizing enzymes. CYP2B6 is the only significant enzyme in the CYP2B subfamily and although it accounts for <10% of the total human hepatic P450 content, it metabolizes numerous substrates for a variety of clinical conditions. CYP2B6 is of special importance in the South African context as it is required for the metabolism of two of the drugs used in first line ART of HIV/AIDS, efavirenz and nevirapine, as well as the antituberculosis agent rifampicin [44]. 

Genetic variants, such as *CYP2B6:*516G>T and 785A>G, defining the allele CYP2B6*6, have been associated with ADRs of efavirenz treatment and are more common in Africans than in Caucasians [44]. CYP2B6*6 was detected in Coloured and Xhosa patients with HIV [45, 46], and in a Venda cohort [47] at frequencies that ranged from 30-36%. This allele was found at a frequency of 37% in the Batswana [48], but at higher frequencies (49%) in Ghanaians and Zimbabweans [49, 50]. Cohen *et al*. [51] reported that although the CYP2B6*6 allele is common in South African patients on simultaneous rifampicin-based antitubercular therapy and ART (including efavirenz), no dosage adjustments were required. CYP2B6*6 was associated with high efavirenz concentrations and severe sleep disturbance in this cohort. 

#### CYP2C9 and VKORC1

Members of the CYP2C enzyme subfamily, which are responsible for metabolizing approximately 20% of prescribed drugs [52], are also valuable candidates for pharmacogenetic applications. CYP2C9 is responsible for metabolism of drugs such as glipizide, tolbutamide and warfarin, the oral anticoagulant of choice to treat and prevent thrombotic disease in South Africa. Functional alleles that reduce the clearance of warfarin may result in higher bleeding risk and thus require lower dosage, however these alleles need to be considered in combination with *VKORC1 *(vitamin K epoxide reductase complex, subunit 1) variants. The VKORC1 protein is inhibited by warfarin, therefore decreasing the amount of vitamin K available as a co-factor for clotting proteins. Thus, genetic variants affecting VKORC1 will have an impact of warfarin response [53, 54]. Currently, the US Food and Drug Administration (FDA) recommends genetic testing and subsequent dosage adjustments prior to warfarin treatment. The recommended range of expected therapeutic warfarin doses based on *CYP2C9* and *VKORC1 *genotypes, however, usually only includes *VKORC1 *-1639G>A (rs9923231) and 1173 C>T (rs9934438) and CYP2C9*2 and *3 variants, which are prevalent in Caucasian and Asian populations [55]. Unfortunately, specific guidelines based on genotypes prevalent in African populations are lacking. The CYP2C9*5, *6 and *9 alleles were detected in the Venda population [56] while these three alleles, as well as CYP2C9*3, *8 ,*11 and *31 were identified by Mitchell *et al. *[54]. The latter cohort comprised of 213 individuals (including 100 controls and 113 patients taking warfarin) from the South African Black population from the Johannesburg region (no ethnic group specified). Interestingly, CYP2C9*3 and *5 were not detected in a Ghanaian population [57], but *3 was detected in an Ethiopian population [58], while *5 was detected in a Beninese population [59], indicative of the fact that African populations cannot be treated as one unit. Twenty six novel *CYP2C9* variants, as well as three known *VKORC1* variants (rs72547529, rs7200749 and rs7294), were also detected in the South African cohort studied by Mitchell *et al.* [54]. The latter study furthermore determined how 11 *CYP2C9* and two *VKORC1 *variants, present at allele frequencies of ≥ 0.02 in their cohort, influence warfarin dosage variability. It was concluded that the *CYP2C9* and *VKORC1* variants, along with a small subset of environmental factors, account for 45% of warfarin dosage variability. More specifically, it was found that while the FDA approved *CYP2C9 *variants, CYP2C9*2 and *3, occurred at frequencies <0.005 in the African population in South Africa and may therefore be of little value to pharmacogenetic tests in this population, additional variants not included in these guidelines were associated with warfarin variability in this population. Three *CYP2C9 *variants (*8, and two novel SNPs, g16179 and g46028) were associated with decreased warfarin dosage and two *VKORC1 *variants (rs7200749 and rs7294) were associated with increased warfarin dosage and should therefore be included in South African warfarin dosage guidelines. However, before these guidelines are implemented, more genetic variation detection and prospective clinical studies are required, especially because *VKORC1* variants-1639G>A and 1173 C>T, the major determinants of dose variability related to *VKORC1 *[60]*, *were not included in Mitchell’s study [54]. 

#### CYP2C19

Another member of the CYP2C subfamily, CYP2C19, metabolizes drugs used in the treatment of malaria, HIV/AIDS, depression and thromboembolic diseases and genetic variants have been implicated in ADRs and treatment failure [9]. Of these gene-drug interactions, the *CYP2C19*-clopidogrel interaction has recently been ranked as one of the most important for pharmacogenetic applications [61] and individuals carrying the CYP2C19*2 allele are more likely to experience a cardiovascular ischemic event or death [62]. Data on genetic variants in the *CYP2C19* gene in Coloured, Xhosa and Venda populations revealed that the majority of individuals could be classified as IMs [47, 56, 63, 64]. Striking differences in the frequencies of the four CYP2C19 metabolizer classes were noted when comparing Coloured and Xhosa populations to each other, but also to international Asian and Caucasian populations [64]. Important alleles such as CYP2C19*2 and the African specific *9 were detected in the Coloured, Xhosa and Venda populations at comparable frequencies, but *17 has only been genotyped in the Xhosa and Coloured populations. The CYP2C19*17 allele has been associated with an improved response to clopidogrel, but also with an augmented risk of bleeding [9, 65]. The non-functional CYP2C19*3, which is absent in the majority of African populations and present in higher frequencies in Asian populations, was only present in the Coloured population [64], reflecting the high level of admixture in this population. Two novel alleles (CYP2C19*27 and *28) were also identified in the Xhosa and Coloured cohorts in the latter study. *In vitro* studies showed that CYP2C19*27 may cause a decrease in expression of downstream coding sequences. CYP2C19*28 was present at very low frequencies in the Xhosa and Coloured populations, indicating the importance of identifying novel and rare variants in South African populations. 

#### CYP2D6

CYP2D6 is responsible for the metabolism of approximately 25% of all prescribed medications, including anti-depressants, antipsychotics, antiarrhythmics, anticancer agents and opioid analgesics, such as codeine and morphine [66]. The *CYP2D6* gene is known to be highly polymorphic, with variable allele frequencies in different populations [67, 68]. The most comprehensive studies on allelic variation in South African populations were performed in the Coloured [69], Venda [47, 56] and Xhosa populations [70]. Resequencing and genotyping have revealed a rich allelic diversity, including non-functional alleles as well as novel alleles in the Coloured (CYP2D6*64, *65 and *66) and Xhosa (CYP2D6*73 and *74) populations. All three populations had a high prevalence of the reduced function CYP2D6*17 and *29 alleles, explaining in part the high incidence of IMs seen in these and other African populations [56, 71]. Interestingly, both the Coloured and Xhosa control populations exhibited a very high CYP2D6*5 (gene deletion) allele frequency (Coloured = 17.2%, Xhosa = 14.2%), while the frequency of this allele was reported to be 5% in the Venda population [47, 63, 69, 70]. The allele frequencies reported for the Coloured and Xhosa populations also differed notably from the Venda for other alleles, such as CYP2D6*10 (Table **2**), as well as from other African populations [68]. Results from a study by Wright *et al. *[70] showed that at least 12.5% of the Xhosa cohort, representing the extreme ends of the CYP2D6 phenotypic classes, would most likely benefit from pharmacogenetic testing prior to the treatment with medication metabolized by CYP2D6.

#### CYP3A4 and CYP3A5

The CYP3A family of enzymes, including CYP3A4 and 3A5, is involved in the metabolism of more drugs than any other biotransformation enzymes [72]. The CYP3A family is also known for its large inter-individual variation in enzyme expression, due to a combination of environmental and genetic factors [73]. Adult CYP3A activity is largely determined by genetic variants in *CYP3A4 *and/or *CYP3A5*, as active metabolism of the one can compensate for defective metabolism of the other [74]. Numerous drugs are metabolized by CYP3A4, including calcium channel blockers, erythromycin, local anaesthetics and the large majority of non-nucleoside reverse transcriptase and protease inhibitors for ART [75]. The best characterized variant in *CYP3A4* is probably the -392A>G polymorphism, the key variant of haplotype CYP3A4*1B. The frequency of the -392G allele in indigenous South African populations from the KwaZulu-Natal (mainly Zulu [76]) and Western Cape provinces (Xhosa [46]) (see Fig. **1**, Table **2**) is similar to that of other African populations (69-82%) [77-79]. Chelule *et al.* [76] also determined the frequency of this variant in Indian (11%) and Caucasian (57%) ethnic groups from the KwaZulu-Natal province of South Africa. The frequency reported for this Caucasian cohort was much higher than that reported for a Caucasian population from the Western Cape province [80] and other Caucasian populations, i.e. 2-10% [72, 81]. The frequency of CYP3A4*1B reported for the Coloured population varied from 21.2% [80] to 57.1% [46]. Discrepancies in allele frequencies reported for the Coloured population may illustrate different percentage contributions from the ancestral populations [34, 36], and will always complicate the use of an average population frequency for many genetic variants in this population. Fernandez *et al*. [80] also noted that the variant -392G-allele was associated with an increased susceptibility to developing prostate cancer in both Coloured and Caucasian cohorts from the Western Cape, similar to findings in the African American population [82].

CYP3A5 is the major P450 enzyme in the esophagus, and polymorphisms in *CYP3A5 *have been proposed as risk factors for esophageal cancer, which is highly prevalent among African males in South Africa [83]. CYP3A5*3, resulting in severely decreased enzymatic activity, was detected in the Xhosa, Coloured and Caucasian populations at markedly different frequencies [80, 84] (Table **2**). Another allele, CYP3A5*6, which includes a functionally significant polymorphism giving rise to absence of activity, is the most common defective allele in the African (Xhosa) population, but is less prevalent in the Coloured population [84] (Table **2**). The frequency of CYP3A5*3 in other African populations is 12-15%, and the frequency for CYP3A5*6 is 14-16% [78, 79]. The African specific allele, CYP3A5*7, is rare in the Xhosa and Coloured populations [84]. Notably, the frequency of *3 was significantly higher in the Coloured control population, compared to oesophageal cancer patients from the same population group (*P*=0.025) [84], while no statistically significant difference in allele frequencies was seen between controls and patients with prostate cancer from this population [80]. 

### 3.2 Other Pharmacogenes

#### ABCB1

*ABCB1* (*MDR1*), member 1 of subfamily B of the adenosine triphosphate-binding cassette (*ABC*) genes, encodes P-glycoprotein (P-gp). P-gp exhibits broad substrate specificity and plays an important role in the trans-membrane transport of numerous therapeutic drugs [85]. Furthermore, P-gp is clinically relevant due to its central role in: (**i**) multi-drug resistance, and (**ii**) drug-drug interactions [86]. There is significant variability in the *ABCB1* SNP and haplotype frequency distribution among different ethnic populations [85]. Although association between genetic variation and phenotype (including P-gp expression, activity and drug response) remains largely inconclusive [87], genetic profiling of this important pharmacogene should eventually be considered in pharmacodiagnostic tools. 

Allele frequencies for the most extensively studied polymorphism in the *ABCB1* gene, 3435C>T, vary substantially between Caucasians (50%) and Ghanaians (10-11%) [79, 88]. Varied frequencies are also evident in South Africa for African, Coloured, Indian and Caucasian populations [46, 76] (Table **2**). While *ABCB1* variants, 1236C>T and 2677G>T/A, were not detected in Ghanaians [79], these two variants, as well as the -129T*>*C variant, were detected in HIV patients of the Xhosa and Coloured populations [46]. These authors found a significant association between the -129T>C and 2677G>A genotypes and immune recovery in response to ART [46]. 

#### NAT1 and NAT2

Genetic variants in N-acetyltransferase (*NAT*) genes, such as *NAT1* and* NAT2*, affect specific activity of the encoded enzymes that results in slow or rapid acetylation of the drugs that they metabolize, leading to toxic side effects or drug inefficacy. A total of 25.7% putative slow *NAT1 *genotypes were detected in 101 African individuals, the majority of which were Tswana speaking from the North West province [89]. This cohort also displayed high genetic diversity for *NAT2*, which corresponds to *NAT2* genotyping studies in the Venda population [47, 90]. The frequencies of the four slow acetylation *NAT2* alleles, *5, *6, *7 and *14 were lower in the Xhosa population, compared to the Venda population, with the African specific NAT2*14 being fourfold more prevalent in the Venda than in the Xhosa [90, 91] (Table **2**). A new allele, NAT2*6E, was also reported at a frequency of 8% in the Venda population, corresponding to the frequencies found in Zimbabweans and Tanzanians [90]. Rapid *NAT1 *and *NAT2* genotypes were present at a significantly higher frequency in the African population from South Africa compared to a Caucasian cohort from the United Kingdom (UK) [89]. The authors consequently assumed that the significant differences in *NAT* genotypes could contribute to the distinctive cancer morbidity and mortality pattern seen in the African populations in South Africa. Furthermore, in a review paper on *NAT2* polymorphisms and their effect on isoniazid treatment in TB patients, the role that slow acetylation alleles could play in patient compliance, isoniazid-toxicity and the emergence of drug-resistant strains of mycobateria, is emphasized [14].

#### TPMT

TPMT (thiopurine S-methyltransferase) is responsible for catalyzing the S-methylation of thiopurines such as 6-mercaptopurine and azathioprine [92], which are immuno-suppressant drugs. The value of this gene for pharmaco-genetics is highlighted by the fact that it was one of the first genes for which guidelines for dosing were given by the Clinical Pharmacogenetics Implementation Consortium [92]. Additionally, a recent study has found a compelling association with the *TPMT *variant, rs12201199, and cisplatin-induced hearing loss [93]. Heckmann *et al*. [94] investigated the relationship between azathioprine toxicity and *TPMT *genotype in a South African cohort and reported that heterozygosity of TPMT*3A and TPMT*3C was significantly associated with haematological toxicity (*P*=0.012) and hepatotoxicity (*P*=0.005). Of the three alleles investigated, TPMT*2 was absent in all three of the South African populations (African, Coloured and Caucasian) and TMPT*3A and TPMT*3C were present at very low frequencies. Interestingly, investigation of TPMT activity showed differences between the ethnic groups, such that the African controls exhibited lower activity than the activity previously reported for African Americans, which was similar to that of the Coloured individuals. These findings correlate to the fact that both the South African Coloured and African American populations are highly admixed and may have similar ancestry components. With regard to cisplatin-induced hearing loss, although no studies examining the associated variant have been reported in South African individuals, the frequency of rs12201199 is ten times higher in Yoruban individuals from Nigeria when compared to Caucasian individuals from Utah [95], therefore further examination is warranted in African individuals.

From the examples discussed here, it is clear that the limited number of South African populations studied to date display distinctive genetic profiles, emphasizing the requirement for appropriate genotyping platforms to adequately capture the genetic diversity in each population. It is already evident that some of the populations, such as the Xhosa and Coloured populations, need to be considered independently. Preliminary data on these two populations analyzed with a custom pharmacogenetic SNP array has further substantiated this finding for other genes, not discussed in this section [42]. However, more extensive genetic diversity studies in geographically/culturally isolated populations are warranted to determine to what extent the different African populations differ from each other (*e.g.* Nguni from the Sotho language group). Often studies simply refer to a “Black South African” or “African” populations without specifying the specific ethnic group. Likewise, data for different South African populations, including African, Coloured, Caucasian and Asian/Indian individuals are often pooled [96]. From the examples given in the preceding section, it is clear that such studies do not contribute adequately to the identification of the unique genetic profiles of our diverse populations. Knowledge of the full spectrum of genetic diversity in these populations is a prerequisite for clinical application of pharmacogenomics in South Africa. Estrella *et al*. [97] have illustrated that “skin color correlates poorly with genetic ancestry and, by extension, poorly represents the pharmacogenetic diversity” within the Brazilian population, with African, European and Amerindian ancestry. Often this is also true for our populations, especially for the Coloured population, where significant variation in ancestry proportions between individuals is evident [34, 36]. These unique genetic profiles bring unique challenges, but also unprecedented opportunities for developing and implementing pharmacogenomic testing and novel drug development.

## 4 THE NEXT PHASE OF PHARMACOGENOMIC RESEARCH IN SOUTH AFRICA 

Future South African genomic research, including studies of pharmacogenomic nature, should focus on the diagnosis, prevention and treatment of the major communicable and non-communicable diseases in the country. Such research needs to be carefully planned to enable the efficient use of limited funding and thus help address the disparity in the number of genomic studies that have already been performed in this and other African countries [98]. As discussed in the preceding sections, most of the pharmacogenetic research in South Africa has been performed on single candidate genes and baseline allele frequencies have been determined for a handful of pharmacogenes in a few populations. Future studies focusing on the country’s populations should aim to bring the field into the genomic era of research. In order to achieve this goal and to ensure sustainable research in pharmacogenomics in South Africa, particular attention should be given to three key areas with regards to genomic study design: (**i**) the extension of existing and the establishment of new national and international multi-disciplinary collaborations, consortia and research networks, (**ii**) the collection and storage of relevant biological samples in bio-repositories, including thoroughly assessed clinical and demographic data, and (**iii**) the careful evaluation of strategies and methodology that should be used in order to ensure novel research that contributes to the field on a global level.

The advent of genomic technologies, such as DNA micro-arrays and next generation sequencing, has revolutionized the number of genetic variants/regions that can be studied in a short amount of time in a single study. However, with this increased number of associations comes the substantial problem of multiple testing [*e.g.,* *P *values of <5x10^-8^ are generally required for significance in genome-wide association studies (GWAS)]. This is especially problematic for genomic studies of complex traits and diseases, where the effect size of individual genetic polymorphisms is likely to be small [99]. Cohorts of thousands of samples are required in such studies in order to obtain adequate statistical power to detect positive associations. It is therefore important to form strong collaborations with fellow research groups, especially those on the African continent, to help in the assembly of large cohorts of patient samples and population matched controls. This is an area that has been inadequately addressed in the past, with poor communication between African research groups. From a pharmacogenomic point of view, collecting cohorts of patients on the same treatment regime is particularly challenging and sometimes requires the collection of samples from numerous study sites. Setting up research consortia and networks similar to the National Institutes of Health (NIH) Pharmacogenomics Research Network [100] would make the organization of such studies more streamlined. This would also ensure that strong proposals can be drafted and subsequently used to provide research that is relevant to the South African community and other African populations. 

The collection and storage of biological specimens in bio-repositories is imperative for efficient genomic research, as well as for the future of personalized medicine. Not only do bio-repositories aid in generating large numbers of samples for these types of research, but they also increase collaborative potential, as resources can be combined or shared nationally and internationally between bio-repositories [101]. Matimba *et al*. [47] reported on an initiative to establish a biobank of samples from African populations and emphasized its potential for pharmacogenomic applications by reporting on pharmacogenetically relevant alleles in nine ethnic groups from five African countries. Only one South African population group, the Venda, was included in this preliminary report. Future endeavors should attempt to perform comprehensive sampling of the diverse South African populations, and should include patient cohorts.

Large scale genomic studies in South Africa should analyze phenotypes that place the highest burden on local health care systems, such as those discussed in preceding sections. Findings from these studies could stratify particular local populations into groups or subpopulations that share a particular genomic risk marker, which could receive a different therapeutic intervention. Additionally, it is important that there are clear guidelines for the assessment of phenotypes before sample collection commences. A clear illustration of this can be found in GWAS of type 2 diabetes. Genetic variation in the *FTO *(fat mass and obesity-associated) gene is a confirmed risk factor for the development of type 2 diabetes, with risk most probably being mediated through body mass [102]. Meta-analyses of type 2 diabetes GWAS data have shown that this association was not detected in studies where obesity was an exclusion criterion for cases (i.e. Type II error) [103]. Standardization of phenotype is therefore important for future studies in South African populations to prevent phenotypic heterogeneity and allow for the generation of robust findings. 

To avoid potential pitfalls, genomic research in genetically unique and diverse South African and African populations should involve a distinct set of methodological considerations. The type of platform used to perform research is of particular importance in this regard. Although commercial genotyping arrays are improving with regard to the amount of genome-wide variation that is captured, they should still be used with caution in African populations. In the same way that incomplete clinical data can affect the results of GWAS, incomplete allele coverage can have a comparable effect [104]. There are a number of commercial arrays available that have been designed especially for pharmacogenomic applications [*e.g.,* AmpliChip (Roche) DMET (Affymetrix), ADME core (Illumina)], yet even these platforms may not be adequate for pharmacogenomic applications in South African populations [64, 70]. Additionally, genotype-phenotype discordance appears to present more frequently in subjects of recent African diaspora when compared to Caucasian individuals and may be partially explained by rare or African-specific genetic variation [56, 71]. Ultimately, however, if genetic variation is adequately captured, African populations offer the advantage of allowing for fine mapping of GWAS signals and the subsequent identification of actual “causal variants”, due to the low LD observed in their genomes [104]. 

The goal of obtaining a thorough catalogue of genetic variation present in African populations is being aided by large scale population-based resequencing endeavors such as the 1000 Genomes Project [105], which will allow for the design of more comprehensive microarrays. Unfortunately, no genomes from the United Nations sub region, “southern Africa”, are currently included in the 1000 Genomes Project or the HapMap3 Project [106]. Sequencing technologies can be effectively used in South African populations to capture the unique genetic variation present in these individuals. Limited targeted resequencing of candidate pharmacogenes has often been used to reveal unique genetic variants [56, 64, 70], while whole genome sequencing results are also available for one South African genome [28].

Next generation sequencing already has applications in pharmacogenomic research [107], and by implementing the capture of targeted regions such as the exome (~180 000 exons), rapid and cost effective alternatives to genome sequencing are being provided. Investigating the coding regions allows for the identification of polymorphisms that have a large effect on protein function and therefore represent relatively low hanging fruits in this type of research. Current pharmacogenomic studies that employ such technologies should perhaps select samples at the extreme ends of the phenotypic spectrum to improve the chances of finding positive associations, with relatively strong effect sizes. However, as the cost of sequencing rapidly decreases [108], analysis of large numbers of whole genome sequences, which are the gold standard in genomic research, should become financially viable in the near future. 

Finally, since the majority of diseases that require urgent attention in South Africa are complex disorders, often entailing multi-facetted treatment, it is important that high standard genomics studies are performed so that they can be integrated into holistic or systems biology approaches. Such approaches would entail the simultaneous consideration of the various ‘omic’ disciplines (*e.g.,* transcriptomics, proteomics, metabolomics and epigenomics) to elucidate novel pathways for therapeutic targets as well as to shed light on gene networks associated with disease and/or drug response [109, 110]. This highlights the inadequacies of the majority of candidate gene studies and demonstrates that future genomic studies need to accurately dissect the intricate nature of complex disorders in order to facilitate translational research. 

## 5 ETHICAL, LEGAL AND SOCIAL ISSUES (ELSI)

As argued in preceding sections, the endeavor to include South African populations in future genomic research is vital for the successful application of pharmacogenomics within the country. It is important though that individuals and communities consenting to partake in research are recognized as key partners and not merely as resources for the research. Furthermore, they should be protected from potentially negative outcomes emanating from these studies. 

Ethical and social challenges encountered with genomic/pharmacogenomic research in South Africa are very similar to those encountered by other developing countries [111]. Practical issues such as language barriers should also be considered, and to enable proper communication between researchers and participants, the latter should receive comprehensive information and informed consent documents in their home language, particularly if they are not fluent in a second language. Furthermore, researchers should be sensitive and respectful to social and cultural differences between communities and should incorporate the customs and beliefs of the participants into their research design [112]. 

To quote Nhlanhla Mhkize [113], “Investigators, therefore, must understand that traditional conceptions of medicine and disease employ dual understandings”, including both “physical and psychic components”. In South Africa it has been estimated that approximately 80% of the population visit the 200,000 to 350,000 traditional healers practicing in the country [114]. Although indigenous medicine may be perceived to conflict with “western” medicine, traditional healers are a highly valuable asset to the country. Researchers from the University of KwaZulu-Natal have initiated a program to train traditional healers in HIV prevention, counseling and care [115]. Thus, by harnessing the abilities of traditional healers to bridge cultural gaps between western and African customs and medicine, a much needed and trusted source of emotional/spiritual care for terminally ill patients is provided, which works in unison with a western therapeutic approach. By using regional social structures and systems as channels of communication between researchers and participants, community engagement is encouraged. This should also help to create a “sense of research ownership by the communities” [116]. Although much can be learnt from projects such as HapMap 1/2/3 [106] and MalariaGen [117] regarding the ELSI surrounding genomics research in a developing world context, every research project will bring its unique challenges, and ELSI should therefore feature strongly on the agenda of any genomic/pharmacogenomic research project.

As has already been highlighted by this article, South Africa is a country which is rich in cultural and genetic diversity. However, coupled with the obvious benefits associated with this diversity, is the potential for exploitation, particularly since the human and technological resources that are required to analyze this material on a grand (genomic) scale still remain underdeveloped in the country. This situation is aggravated by limited national legislation governing the harvesting, storage, analysis and export of human genetic/genomic material [118]. 

The notion of genomic sovereignty is rapidly gaining prominence in the local and international community and its inclusion in both local and international legislation is seen as an urgent requirement to prevent exploitation of the populations that are being researched by those from outside the country. Genomic sovereignty is the capacity of a people, a country or nation to own and to control both access to and use of, samples, data and knowledge concerning or emanating from genomic material [119]. This definition pertains to genetic material of human, animal and plant origin. Two areas have been given particular attention, namely access and benefit sharing.

The notion of access defines the need for local populations to have access to data that is generated from their genetic/genomic material. The disease profile in South Africa is characterized by very specific ethnic/racial differences, including for example diabetes, obesity, hypertension and hyperlipidemia [7, 120-124]. Since the combination of local environmental conditions, coupled with genetic predisposition to certain diseases, will impact on the incidence/prevalence of the disease, it goes without saying that any genetic/genomic data that emerges from studies on indigenous populations should be made available to those populations to allow strategies for prevention, diagnosis and treatment of the relevant diseases to be developed. This in turn will affect public health policies and the attribution of national/health budgets in a more cost-effective manner.

The notion of benefit sharing requires that some form of financial or social benefit is returned to the population from which the genetic material was derived [125]. Although benefit sharing arrangements may be complex and challenging, it is essential that both the communities and their governments are involved in these discourses [126]. In South Africa, benefit sharing is covered in the case of plants and animals in Chapter 6 of the Biodiversity Act (no. 10 of 2004) [127]. However, there is at present no reference in any South African legislation to the notion of benefit sharing (or to access for that matter) regarding human material. For many years, genetic material has been leaving South Africa to be analyzed outside of the country. In many cases, neither the principles of access or benefit sharing have been respected. With regard to the former, once data derived from this material is published in the public domain, it does become accessible. However, this is by default, and no specific attention is given to returning it to the community from which it was derived. This is particularly important when drug clinical trials have a significant pharmacogenomic component. In this case, a large amount of very important information is generated in laboratories outside the country, and very rarely does this find its way back into the country to be utilized for the benefit of the indigenous populations from which it was derived. Benefit may take the form of financial benefit, social benefit (housing, schools, healthcare facilities, *etc*.) and also scientific capacity development.

What are the solutions to these problems? First, the notion of genetic sovereignty needs to be debated more widely in open public *fora* – both nationally and internationally. Lobby groups need to be formed to ensure that exploitation is prevented and that the principles of access and benefit sharing are respected. 

Second, measures need to be put into place to regulate and monitor the flow of genetic/genomic material into and out of the country. Despite the current absence of specific legislation in this regard, regulation and monitoring can be achieved in two ways. This includes; (**i**) Research Ethics Committees insisting that these matters are given attention in projects that involve human genetic/genomic material, and (**ii**) the National Department of Health insisting on the same requirements in export permits that are issued for material that leaves the country. Currently applications for such permits require very little information. 

Third, human capacity development is a critical issue that could be addressed as part of a benefit-sharing arrangement with companies/institutions/individuals that work on South African genetic/genomic material beyond our borders. One of the areas that requires particular attention is the development of bioinformatics skills, in order to address the computational and statistical bottlenecks associated with the analysis of the vast amounts of data generated from genomic research, which is seen as a national priority (National Biotechnology Strategy for South Africa [21]). It is important that locally generated research is used to train South African scientists and that intellectual property is appropriately managed in order to build the required resources within the country. 

Fourth, South Africa is currently revising the section (Chapter 8) of the National Health Act (no. 61 of 2003) which deals with “Control of use of blood, blood products, tissue and gametes in humans” [118]. Chapter 8 has to date not been promulgated, and this part of the legislation is currently covered by the largely outdated Human Tissue Act (no. 65 of 1983) [128]. The current process of revision should be used to address the legislative gap with respect to human genetic/genomic material.

Many of the recommendations listed above are of course transferable to other developing countries that are exposed to similar universal problems of unidirectional flow of information and potential exploitation. Thus, it is hoped that South Africa can in the future serve as a reference for developing countries for the successful implementation of ethical, legal and social considerations of genomic research. 

## 6 PHARMACOGENOMIC STUDIES IN SOUTH AFRICA: CHALLENGES AND OPPORTUNITIES IN AN AFRICAN CONTEXT

Although the benefits of the Human Genome Project are beginning to be realized, there has been a skewed distribution in terms of where related research has been carried out. Thus far, the majority of completed genomic research studies (75%) have been conducted exclusively in populations of European descent while very few have been done in Africa or on Africans [98], despite the fact that Africa carries a disproportionately large burden of avoidable diseases, including infectious diseases as well as an alarming increase in non-communicable diseases [8]. Ironically, the high prevalence of communicable and non-communicable diseases in South Africa offers great opportunities for genomic research. Vast numbers of patients from diverse populations are available for epidemiological and clinical studies, also suited for numerous clinical trial designs, which can be combined with genomic or pharmacogenomic studies. The diverse populations of the country offer a variety of advantages. While the high allelic diversity and short areas of LD in African populations allow fine mapping of loci, the highly admixed Coloured and homogenous Afrikaner and Indian populations permit other mapping strategies. Additionally, ideal opportunities for genotype-phenotype correlation studies exist, as the genetically diverse populations can be used to assess the influence of genetic variation on phenotypes in similar environments, as well as in different environments (e.g., rural versus urban). These studies could also provide opportunities for epigenetic studies. 

Examples of research opportunities that are ripe in South Africa as a result of these unique disease cohorts, include the study of genes that are associated with viral infection (and HIV in particular), progression to the AIDS defining illness, overall survival, mother to child transmission, as well as ART response. Outstanding work in this regard is already being done in South Africa for HIV/AIDS (*e.g.,* [129-132]) and TB (e.g. ([133-135]), and a number of large well defined cohorts have already been collected (*e.g.* [133, 136, 137]). Some of these cohorts provide ample opportunities for clinical trails that can also be utilized for pharmacogenomic studies. 

Study design is of paramount importance for the success of any research that may be implemented within South Africa. A recent paper outlined the types of pharmacogenomic design that could provide a good guideline for the types of patient cohorts that need to be assembled in order to generate evidence for pharmacogenomics in clinical practice [138]. For example, the incorporation of pharmacogenomic analyses during preclinical and clinical trials could aid in translating genomic discoveries to health care; verifying the clinical validity and utility of genomic findings is thus another important focus area for future research. Currently there is a paucity of prospective genomic studies as well as large scale randomized controlled trials in South Africa and the rest of the continent, therefore these aspects need to be addressed. 

Many more unexplored opportunities for pharmacogenetics/genomics studies exist [139], of which oncology is one. Cancer is the leading cause of death worldwide [140] and the incidence of this disease is gathering momentum in developing countries such as South Africa. The importance of pharmacogenomic strategies that can be implemented for effective treatment of the disease is substantiated by the number of oncology guidelines from both the FDA and European Medicines Agency (EMA), many of which refer to tumor-specific pharmacogenetics [141, 142]. When referring to the application of cancer pharmacogenomics it is essential to make the distinction between germ-line and tumor-specific mutations. Both are important, but while the former relies on determining the baseline frequencies of variation in different ethnic groups, the latter is dependent on the disease. FDA and EMA clinically validated tumor/disease-specific variants can therefore be applied in local treatment strategies.

One of the main obstacles to large scale genomic or pharmacogenomic studies within South Africa is limited funding for research and development purposes [22]. However, new prospects in this regard have recently arisen with a number of new initiatives, of which three are discussed here, namely the Human Health and Heredity in Africa (H3Africa), the Southern African Human Genome Program (SAHGP) and the Pharmacogenetics for Every Nation Initiative (PGENI). These initiatives aim, among other things, to characterize African genomic variation as well as to determine the baseline frequencies of known variation. The genetic variation detected by the H3Africa and SAHGP will be used to aid in the understanding of disease pathogenesis and thereby improve the ability to diagnose, treat and prevent the diseases and disorders which affect Africans, while the PGENI data will be used for pharmacogenetic applications on a global level. Through the implementation of these initiatives, it is hoped that we can influence public health policy, and also improve the infrastructure, human resources and public awareness in genomic research in Africa. 

H3Africa consists of a consortium of African scientists who, with the assistance of well established international partners such as the Wellcome Trust in the UK and the NIH in the US, aims to bridge the research, expertise and infrastructural genomic gap that Africa faces. This genomic research endeavor realizes that the documentation of human genome variation cannot be completed unless Africa and its genetically diverse people are involved. Therefore, this initiative aims to facilitate the integration of research based on the latest genomic technologies in order to create and sustain a network of African centers which will provide training, research and clinical services, with the ultimate goal of improving health outcomes and research in Africa [143]. 

When considering the execution and application of genomic or pharmacogenomic research in an African context, South Africa is one of the countries that is best equipped to accommodate this research. Besides the high burden of disease which South Africa shares with other African countries, the country has one of the best health systems on the continent, including health statistics, and is regarded as the frontrunner for biomedical research in Africa [144]. Upcoming initiatives can quickly and easily bear fruit in South Africa because only minimal additional strengthening is needed in terms of expertise (bioinformaticists, statisticians, biologists, bio-ethicists) and infrastructure, possibly due to the relatively strong economy and stable administration of the country [143]. The SAHGP was recently launched with the specific goal of understanding DNA variation in southern Africans [145]. This project is aimed at forming collaborations between scientists, government and industry to build capacity for genomic research, to institute sustainable resources for this research, including a bio-repository and database, and to “translate the information and knowledge into improvements in human health”. The issue of genomic sovereignty, including benefit sharing, will also be carefully considered. 

The South African government has shown interest in genomic and biotechnology endeavors and the DST already has a National System of Innovation in place which is involved in a ten year plan, focused on moving South Africa’s economy from resource-based to knowledge-based. One of the grand challenges proposed in this plan is the “farmer to pharma” challenge which aims to place South Africa as a leader in biotechnology and pharmaceutical industries in the developing world [21].

With regard to genomics education, the Public Understanding of Biotechnology program of the DST and the Human Genome Education Institute, a virtual entity that is involved in bridging the gap between researchers and the lay public by way of offering public seminars on topics with a leaning towards genomics, is already established. Furthermore institutes such as the South African National Bioinformatics Institute (SANBI), to give one example, are in place to provisionally address issues regarding the generation of high throughput genomic data and the bioinformatic analyses thereof.

Of specific relevance to the field of pharmacogenetics is PGENI, which has been set-up with the main objective of integrating pharmacogenetics into public health care for all global populations. PGENI aims to determine the baseline frequencies of known DNA variants present in 154 genes which are involved with the action of 206 drugs. Populations from 104 countries around the world will be examined, of which South Africa is one of the nine regional centers and is therefore responsible for facilitating collaborations between the surrounding PGENI countries [146]. This again highlights the promise of South African genomic research.

Projects such as the H3Africa Initiative, the SAHGP and PGENI should play an integral role in the coordination of genomic research in South Africa, but also other African countries, by providing infrastructure and capital to local researchers, as well as providing aid in addressing the computational and statistical bottlenecks encountered at present. 

## CONCLUSIONS AND OUTLOOK

South Africa has a turbulent history and has witnessed numerous struggles, but the current battle against the *force* *majeure* of communicable and non-communicable diseases is one of the fiercest yet, placing a heavy burden on its people. Furthermore, to date, drugs have primarily been designed to suit the specific needs of Caucasian and Asian populations and only 1% of the newly developed drugs are focused on the treatment of disease affecting poor countries [147]. In this paper we have argued that the implementation of pharmacogenomic research may play a valuable role in refining current treatment regimes in the country. It may be debated that such research is “too late” for South Africa, or that it is too technologically advanced and not cost-effective. However, with the exciting and promising advent of high-throughput sequencing and new initiatives such as the SAHGP and H3Africa, it seems more likely that not only are we “just in time” [16], but we could assume a place in the global genomic research community. 

In order for pharmacogenetic/genomic biotechnologies to be successful in South Africa, the highly diverse populations making up the Rainbow Nation need to be comprehensively characterized, and research, which has previously focused on candidate genes, needs to move into the genomic arena. However, before South Africa embarks on its pharmacogenomic venture, there are several aspects that need to be addressed and accompanied with appropriate guidelines. Amongst others, the establishment of extensive bio-repositories will require clear guidelines that will ensure that participants are informed and protected, such that data is only utilized for the specified research purposes [148]. Furthermore, the development of extensive multi-disciplinary collaborative networks will ensure that samples in bio-repositories are utilized to their full potential. Such collaborations need to consider the benefit of all participants, but should also be aimed towards capacity building and take genomic sovereignty into account. Researchers should preferably take a holistic view on personalized medicine and should move beyond a purely genetic approach to include all aspects of treatment response, incorporating initiatives such as the Human Proteome Project [149] into research projects. Additionally the manner in which results are reported is equally important, as reliable and complete results can be used for reviews and meta-analyses and ultimately create a strong evidence base for translation into the clinical setting [150]. This is essential as only 3% of global genomic research moves beyond gene discovery [151]. Downstream applications derived from research may include diagnostic tests and development of new therapeutics. However, it is vital that these outputs benefit the South African public and promote local research, while taking intellectual property rights, ELSI and cultural aspects into consideration [152]. In addition to expert knowledge, public engagement in pharmacogenomics is another important aspect, for example, as a means to bring together different forms of knowledge and “ways of knowing” to the fore, including tacit and local knowledge from the citizens and patients [153-155]. Although it is clear that much needs to be done before pharmacogenetics/genomics is successfully implemented within the South African context, success in this domain will not only contribute to the country’s knowledge-based economy, but will also enhance its position as a global research and development partner. 

In the past, South Africa has surprised the world on more than one occasion by making the seemingly impossible, possible. Hopefully, in the future, we will be able to look back at pharmacogenomics in a similar way, as we move towards building a viable and successful national pharmacogenomics program. Success in this arena will also allow South Africa to act as a model for building capacity in the rest of Africa, to aid in the much needed improvement of patient treatment throughout the continent. 

## Figures and Tables

**Table 1:** Distribution of South African Home Languages within the Country, Based on the 2001 Population Census* [32]

Official South African Languages	Number of First Home Language Individuals	Percentage of Total South African Population
IsiZulu	10,677,305	23.8
IsiXhosa	7,907,153	17.6
Afrikaans	5,983,426	13.3
Sepedi	4,208,980	9.4
Setswana	3,677,016	8.2
English	3,673,203	8.2
Sesotho	3,555,186	7.9
Xitsonga	1,992,207	4.4
SiSwati	1,194,430	2.7
Tshivenda	1,021,757	2.3
IsiNdebele	711,821	1.6
Other	217,293	0.5

* The 2001 South African population census is the last census available.

**Table 2:** Frequency Comparisons of Clinically Relevant Alleles Detected in South African Populations

Gene	South African Population		Clinically Relevant Alleles						Reference
Cytochrome P450 Genes									
*CYP2B6*			*6						
	African	Venda (n=81)	0.36						[47]
		Xhosa HIV patients (n=112)	0.32						[46]
	Coloured	HIV patients (n=70)	0.30						[46]
*CYP2C9*			*2	*3					
	African	Unspecified (n=100)	0.00	0.01					[54]
		Venda (n=9)	0.00	0.00					[56]
*CYP2C19*			*2	*3	*17				
	African	Venda (n=81)	0.21	0.00	NG				[47]
		Xhosa (n=100)	0.21	0.00	0.10				[64]
	Coloured	(n=75)	0.17	0.07	0.14				[64]
*CYP2D6^1^1*			*2xN	*4	*5	*10	*17	*29	
	African	Venda (n=81)	NG	0.03	0.05	0.12	0.24	0.06	[47]
		Xhosa (n=53)	0.03	0.04	0.14	0.02	0.13	0.13	[70]
	Coloured	(n=99)	0.01	0.07	0.17	0.03	0.13	0.05	[69]
*CYP3A4*			*1B						
	African	Mainly Zulu (n=110)	0.84						[76]
		Xhosa HIV patients (n=112)	0.78						[46]
	Caucasian	(n=141)	0.04						[80]
	Coloured	HIV patients (n=70)	0.57						[46]
		(n=146)	0.21						[80]
	Indian	(n=103)	0.11						[76]
*CYP3A5*			*3	*6	*7				
	African	Xhosa (n=142)	0.14	0.21	0.01				[84]
	Caucasian	(n=141)	0.94	NG	NG				[80]
	Coloured	(n=99)	0.59	0.12	0.00				[84]
**Other Pharmacogenes**									
*ABCB1*			**1236C>T**	**2677G>T/A**	**3435C>T**				
	African	Mainly Zulu (n=110)	NG	NG	0.14				[76]
		Xhosa HIV patients (n=112)	0.13	0.03	0.10				[46]
	Coloured	HIV patients (n=70)	0.24	0.18	0.21				[46]
	Indian	(n=103)	NG	NG	0.58				[76]
*NAT1*			*3	*4	*10	*11,*14-*16			
	African	Mainly Tswana (n=101)	0.01	0.49	0.51	0.00			[89]

In those cases where the same population was examined by more than one study, the frequency data from the most comprehensive study was used.

1 For those studies which genotyped the *CYP2D6* duplication, those duplications other than

* *2xN* were pooled with the relevant single copy alleles. NG: Not genotyped.

## ABBREVIATIONS

ABC: = Adenosine triphosphate-binding cassette
ADRs: = Adverse drug reactions
ART: = Anti-retroviral therapy
CNV: = Copy number variation
CYP: = Cytochrome P450
DST: = Department of Science and Technology
ELSI: = Ethical, Legal and Social Issues
EM: = Extensive metabolizers
EMA: = European Medicines Agency
FDA: = Food and Drug Administration
GWAS: = Genome-wide association studies
H3Africa: = Human Health and Heredity in Africa
HIV/AIDS: = Human Immunodeficiency Virus/Acquired Immunodeficiency Syndrome
IM: = Intermediate metabolizers
LD: = Linkage disequilibrium
mtDNA: = Mitochondrial DNA
NAT: = N-acetyltransferase
NIH: = National Institutes of Health
P-gp: = P-glycoprotein
PGENI: = Pharmacogenetics for Every Nation Initiative
Pharmacogene: = Pharmacogenetically relevant gene
PM: = Poor metabolizers
SAHGP: = Southern African Human Genome Program
SANBI: = South African National Bioinformatics Institute
TB: = Tuberculosis
UK: = United Kingdom
UM: = Ultrarapid metabolizers
US: = United States
